# Neoadjuvant Therapy With Cabozantinib as a Bridge to Liver Transplantation in Patients With Hepatocellular Carcinoma (HCC): A Case Report

**DOI:** 10.3389/frtra.2022.863086

**Published:** 2022-05-06

**Authors:** Hiral Bhardwaj, Danielle Fritze, Daniel Mais, Venkatesh Kadaba, Sukeshi Patel Arora

**Affiliations:** Mays Cancer Center, University of Texas Health San Antonio, San Antonio, TX, United States

**Keywords:** hepatocellular carcinoma, liver transplant, bridge therapy, cabozantinib, sorafenib

## Abstract

Liver transplant (LT) is the treatment of choice for unresectable, localized hepatocellular carcinoma (HCC). However, transplant is not recommended for patients who have extensive tumor growth and do not meet specific criteria. For these cases, “bridging” therapies are often used to either downstage or prevent tumor progression while patients are on the transplant list. Various pre-transplant therapies have been used, including transarterial chemoembolization, radiofrequency ablation, and systemic therapies. Sorafenib is a well-known systemic agent used for HCC, but research is limited on its use as well as the use of newer agents as bridging therapy. Prospective studies are also lacking. We discuss cases of two patients diagnosed with HCC and treated systemically with cabozantinib prior to transplant without treatment-related complications. This suggests that cabozantinib could be safely used after sorafenib therapy to control disease related to HCC while awaiting liver transplantation.

## Introduction

Transplantation is the only therapy that offers cure for both the HCC and the patient's underlying cirrhosis. However, donor shortage remains a problem and the average wait time for a liver is 6–12 months (1). Due to this long wait time, around 20–30% of patients are dropped from the list due to tumor progression (1). While on the waitlist, patients must maintain a baseline AFP under 500 nanograms/milliliter (ng/ml) to qualify for standard HCC exception points (2). Other risk factors for dropout from the waiting list were listed in a 2013 study as 1 tumor of 3–5 centimeters (cm), 2–3 tumors, lack of complete response to first loco-regional therapy, and high AFP after first locoregional therapy (LRT) (1). Neoadjuvant treatments, or bridging therapies, have been used in order to prevent tumor progression and maintain patient status on the waitlist (3). These bridging therapies include LRT, such as transarterial chemoembolization (TACE), transarterial radioembolization, radiofrequency ablation (RFA), stereotactic body radiotherapy (SBRT), and systemic treatment (3).

In this report, we investigate the role of systemic treatment as a bridging therapy to LT. Sorafenib predominantly targets vascular endothelial growth factor receptor (VEGFR) pathways in HCC. It has been widely used as a standard of care systemic therapy for HCC and has shown to improve survival and time to progression (4). However, its success as bridging therapy to LT has been undetermined. A 2010 study on the use of sorafenib in HCC patients prior to LT was performed with patients ranging from having no side effects to having skin reactions and diarrhea, which required dose reductions or brief discontinuation of the medication (5). The study concluded that sorafenib did not have “extremely negative” side effects during the postoperative course. Of these four patients, only one required re-transplantation 5 days after LT due to hepatic artery thrombosis. None of the patients showed signs of local recurrence or distant metastases at the time of follow-up, although follow-up was short and the authors recognized the need for more data and longer follow-up. Other studies of sorafenib as a neoadjuvant therapy showed, as seen in Table 1, similar 3-year-survival (93% vs. 91%) and recurrence rates (13% vs. 11%) between sorafenib and control groups (7), 0% tumor recurrence in 27 month mean follow-up (9), similar time to progression (71 days vs. 85 days) in sorafenib and control groups (8), sorafenib use associated with intolerability and dose reductions (10), and death rates of 20% in the sorafenib group vs. 8.7% in the control group, but higher rates of acute cellular rejection and early biliary complications in the sorafenib group (6). With similar outcomes between control and sorafenib groups and unpleasant side effects with sorafenib such as skin reactions and diarrhea, its efficacy as bridging therapy is still to be determined.

**Table 1:** Literature review of sorafenib used as a neoadjuvant therapy as a bridge to liver transplantation in hepatocellular carcinoma (HCC).

**Study**	***n***	**Tumor Criteria**	**Average daily sorafenib dose**	**Average sorafenib duration (months)**	**Toxicity**	**Pre-transplant locoregional treatment**	**TTP**	**OS/OS rate**	**Mean follow-up (months)**	**Post-transplant recurrence**
Saidi et al. (5)	4	Milan criteria: 75% in, 25% out	550 mg	3.5	GI, cutaneous, weight loss	TACE: 75% RFA: 25% Resection: 25%	-	100%	-	0%
Truesdale et al. (6)	10	Milan criteria: 90% in, 10% out	560 mg	4.8	GI, cutaneous	TACE: 90% RFA: 10% Radiotherapy: 10%	-	Death rate: 20%	22.6	0%
Frenette et al. (7)	15	Milan criteria: 7% in, 93% out	400 mg	4.8	GI, cutaneous, hyperbilirubinemia, thrombocytopenia	TACE: 80% RFA: 20%	-	93%	12.3	13%
Hoffmann et al. (8)	24	Milan criteria: 100% in, 0% out	-	4.2	GI, cutaneous, thrombocytopenia	TACE: 8.3%	29% progressed, TTP 71 days	-	10.7	0%
Golse et al. (9)	5	Milan criteria: 20% in, 80% out	480 mg	17.2	Cutaneous, severe asthenia	TACE: 40% Resection: 60%	-	60%	26.8	0%
Eilard et al. (10)	12	UCSF criteria: 100% in, 0% out	474 mg	5.2	GI, cutaneous, vision changes, fever, tremor, fatigue, liver enzyme deviation	TACE: 58.3%	TTP: 20 weeks	2-year OS: 83%	40	41.70%

*GI, gastrointestinal; TACE, transarterial chemo-embolization; RFA, radiofrequency ablation; TTP, time to progression; OS, overall survival*.

Cabozantinib is a TKI with molecular targets that include RET, MET, and VEGFR (11). Our literature search did not find any reports of cabozantinib or other tyrosine kinase inhibitors (TKIs) as bridging therapy for transplant. We illustrate two situations where cabozantinib may be used as a systemic treatment for bridge therapy: (1) after progression on sorafenib, or (2) after intolerance to sorafenib.

## Case Presentation

**Figure 1:**
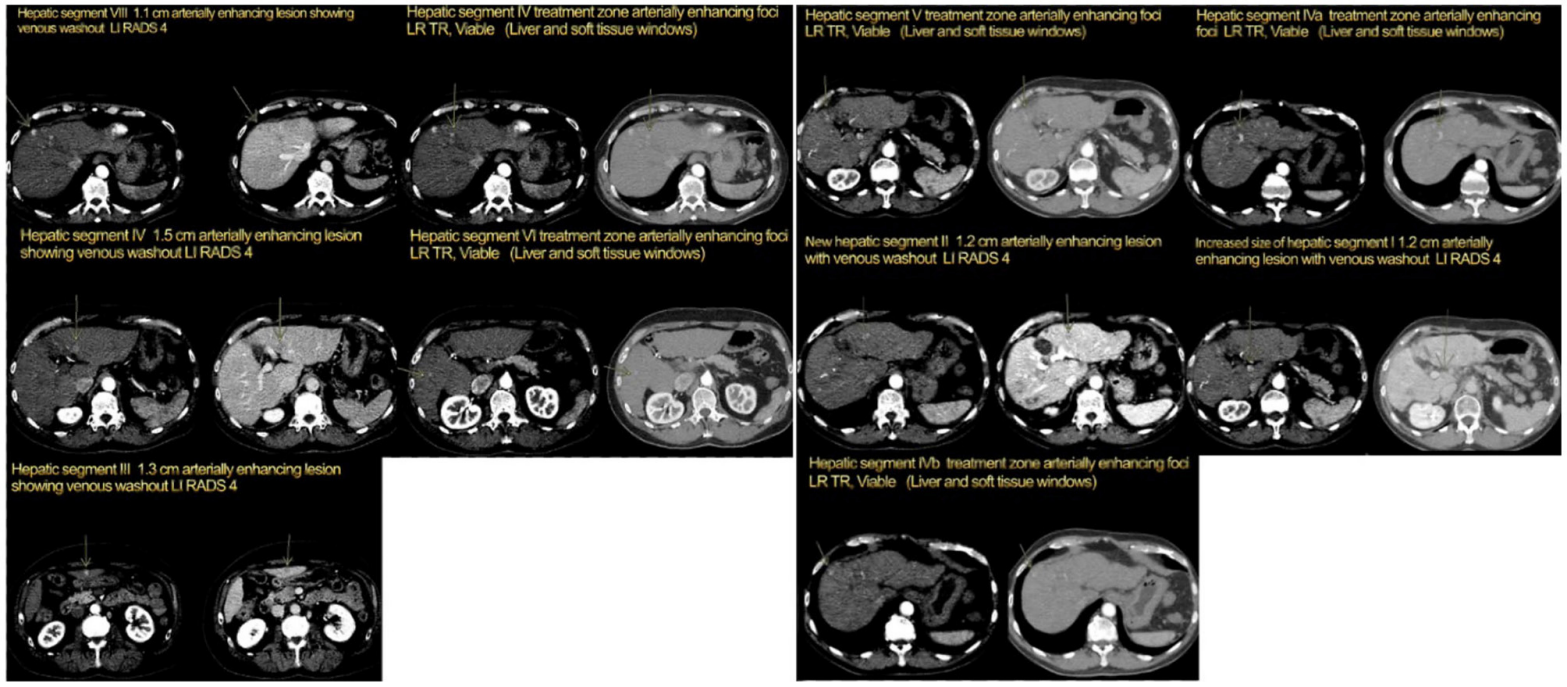
Patient 1, four-phase computed tomography (CT) scan: **Left:** pre-cabozantinib (July 2016), **Right:** post-cabozantinib/pre-transplant (August 2019).

Patient 1 is a 65-year-old male with a history of Hepatitis C Virus (HCV) with sustained virologic response after anti-viral treatment, cirrhosis, and portal hypertension was diagnosed with HCC in February 2015 during surveillance liver imaging. At that time, two enhancing masses consistent with HCC by Liver Imaging Reporting and Data System (LI-RADS) (12) were noted: a 4.2 centimeter (cm) lesion in segment 4A and a 1.1 cm lesion in segment 8, which were LI-RADS 5. Additional smaller lesions did not meet imaging criteria for HCC. The larger HCC was initially treated with trans-arterial chemoembolization (TACE) and the smaller with microwave ablation (MWA) in 2015, resulting in successful downstaging and eligibility for transplantation. While awaiting transplantation, he was found to have residual/recurrent HCC in segment 4A. After multi-disciplinary discussion, HCC was treated with additional TACE, and systemic treatment was recommended as a bridge to transplant. Baseline labs were consistent with Childs Pugh (CP) score B8 (13), HCC stage B using Barcelona Clinic Liver Cancer (BCLC) staging (14), Cancer of the Liver Italian Program (CLIP) score of 1 (14), MELD-Na score of 16 (15), and an Alpha-Fetoprotein (AFP) level of 11.4 ng/ml. Staging computed tomography (CT) chest and magnetic resonance imaging (MRI) abdomen were ordered revealing residual disease (Figure 1).

The patient was started on sorafenib 400 mg twice a day (BID) in October 2016, but this was not well tolerated with the emergence of hand-foot syndrome (HFS). Thus, the dose was reduced to 200 mg BID, and imaging continued to show stable disease until September 2017, when CT abdomen suggested local recurrence (LR-TR Viable). Patient underwent multiple TACE procedures in early 2018 to address the right lobe HCC recurrence.

In October 2018, patient's AFP was 87.1 ng/ml with 2.1 cm residual disease and by November 2018, the AFP had increased to 92.8 ng/ml with CT imaging revealing growth in the segment 8 liver lesion from 9 mm to 1.4 cm (LI-RADS 4). Given progression on sorafenib, the patient was switched to cabozantinib 40 mg daily (QD) (dosing for CP-B per the package insert) and listed on the liver transplant list. He initially developed HFS, and the dose was lowered to 20 mg in January 2019. The patient tolerated this dose well until labs in April 2019 revealed AFP in the 500s with progression on imaging (Segment 8 lesion now LI-RADS 5 and Segment 4 lesion was LR-TR viable; no new lesions). Yttrium 90 radiation (Y90) right and left were performed in April and May 2019, respectively, with an AFP decrease to 77.9 ng/ml on his follow-up in May. Cabozantinib 20 mg QD was continued as no other new lesions were seen (Figure 1). The primary benefit of cabozantinib was likely lack of progression to more advanced disease, like portal vein thrombosis or extrahepatic spread, which would make the patient ineligible for transplant.

**Figure 2:**
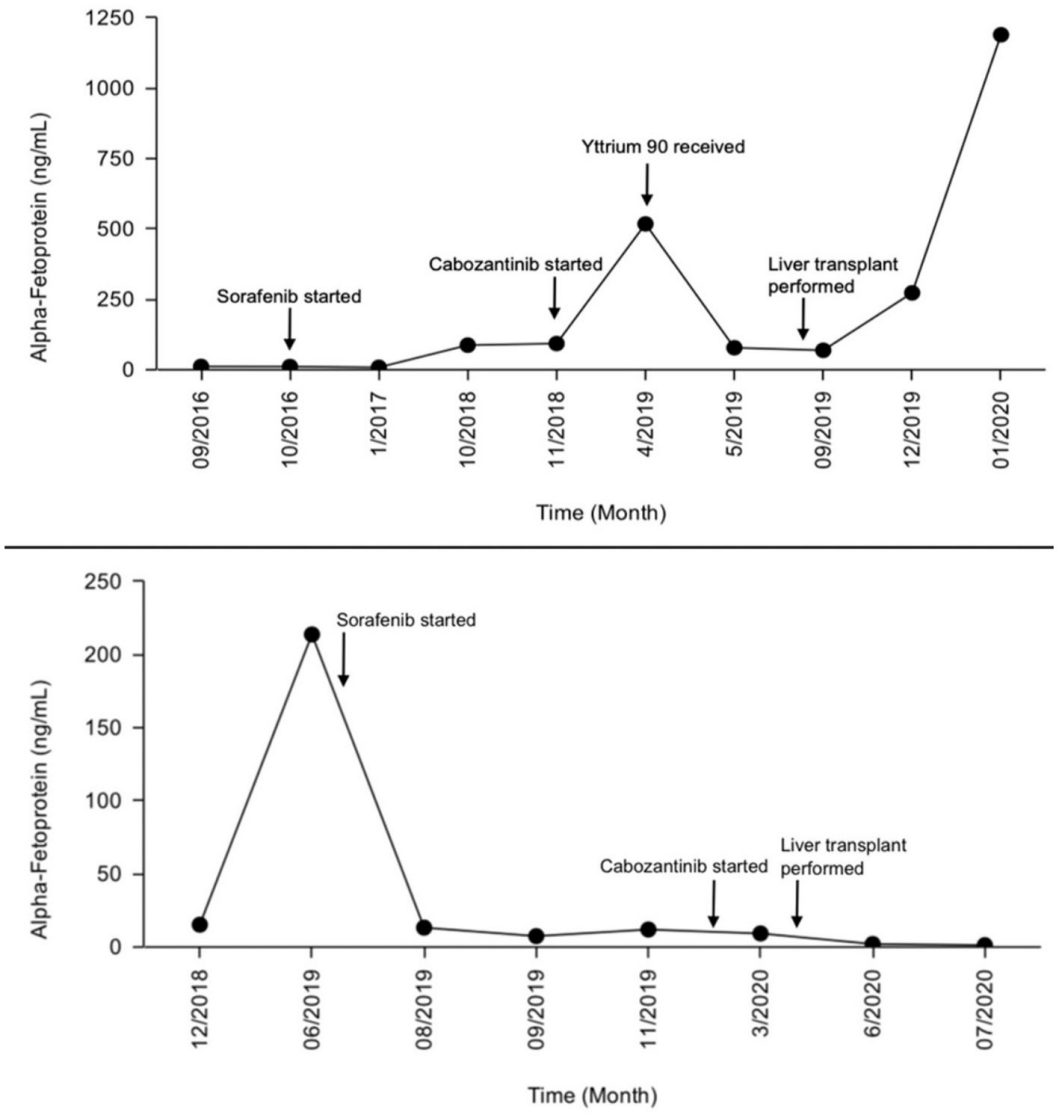
Alpha-fetoprotein (AFP) level progression during treatment (patient 1 top, patient 2 bottom).

The patient underwent liver transplant in August 2019 with explant showing 5 viable tumors, largest 1.5 cm, some with incomplete necrosis, and all with negative margins. Cabozantinib was held, and his AFP was up trending in December 2019 with no new disease on CT (Figure 2). AFP had increased to 1,014 ng/ml on his January 2020 visit with new lung lesion and lytic lesion on T11 on CT chest. Restarting cabozantinib 20 mg QD was recommended and started in February. Disease progression continued with new liver metastasis and AFP up to 21,000 ng/ml in May 2020. Stopping cabozantinib and starting ramucirumab was discussed as well as supportive care with hospice. Patient had a rapid decline in performance status and was transitioned to hospice, dying soon after.

**Figure 3:**
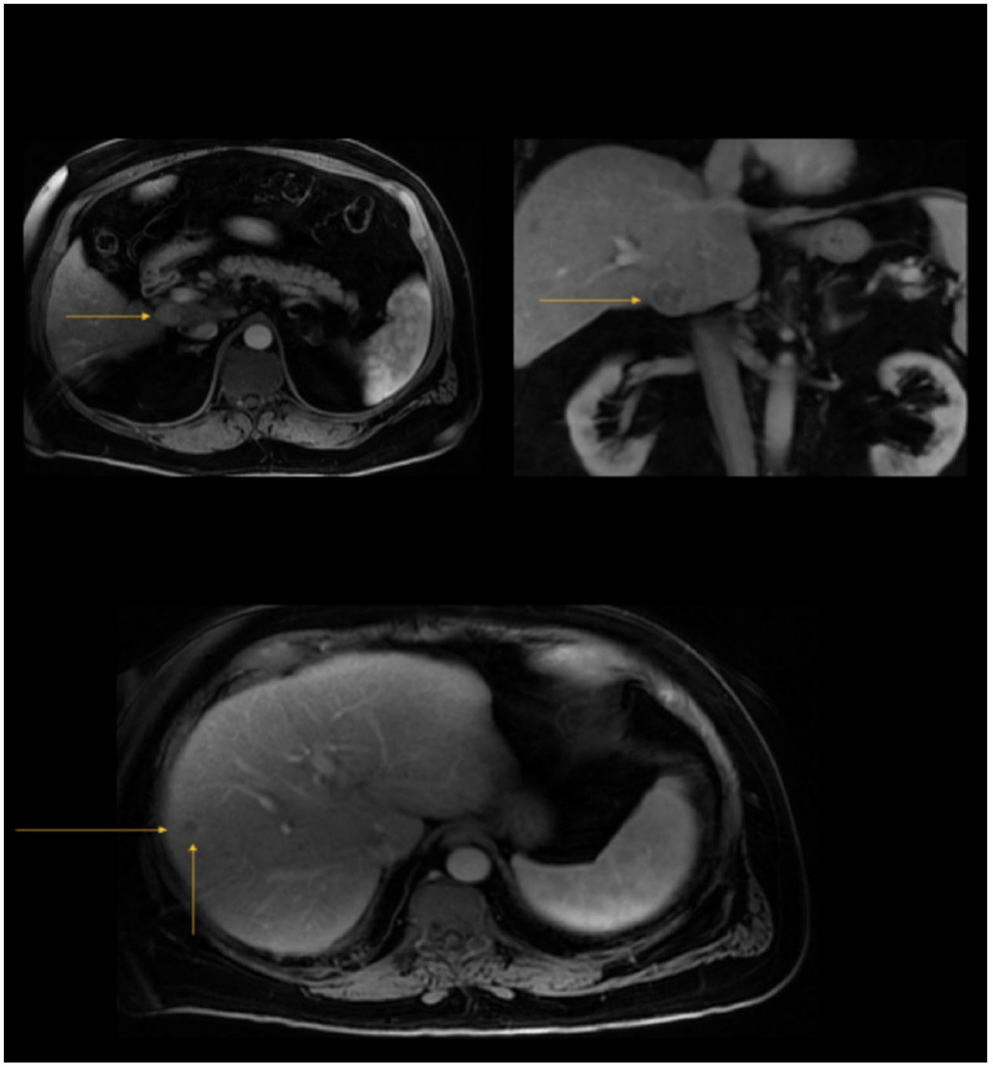
Top: A 2.6 × 2.3 × 2.3 cm arterially hyperenhancing, T2W hyperintense caudate lobe mass demonstrating venous washout and delayed rim enhancement (LI RADS 5) (November 2018, patient 2); Bottom: 1.3 cm poorly defined non-enhancing hepatic segment VIII lesion (LI RADS 3) (November 2018, patient 2).

Patient 2 is a 64-year-old male with history of chronic HCV cirrhosis in SVR after anti-viral treatment since 2016 was diagnosed with HCC in November 2018 on surveillance imaging. Imaging revealed a 2.6 cm caudate lobe lesion LI-RADS 5 (Figure 3) and a 1.3 cm lesion in segment 8, LI-RADS 3 (Figure 3) with BCLC Stage B, MELD-Na Score of 11, and CLIP Score of 0. He presented to our clinic in June 2019 after ablation of the caudate lobe lesion with no residual lesion but slow growth of segment 6/8 lesion at 2.4 cm with an uptrend in AFP (213.8 ng/ml), concerning for progression of multifocal disease. Ablation of the segment 6/8 lesion was performed in June 2019. Due to suspected multifocal progression of HCC and intact synthetic liver function (CP Score A6), multi-disciplinary liver tumor board recommended systemic treatment as a bridge to transplant. Patient had been listed on the liver transplant list in December 2018 following his HCC diagnosis; therefore, immunotherapy was avoided.

He started sorafenib 200 mg BID in July 2019. Patient developed severe diarrhea over the next month on this dose with around 12 episodes per day. Sorafenib was held and resumed multiple times to see if the side effects would resolve, but the patient was unable to tolerate the medication due to diarrhea and HFS. Dose was reduced to 200 mg QD, which was better tolerated. CT scans in August 2019 showed stable disease and AFP had improved in September from the 200s down to 10 ng/ml (Figure 2). Sorafenib was continued as a bridge to transplant with adequate management of diarrhea and HFS with the reduced dose.

The patient developed worsening HFS and diarrhea in November 2019 and plans were made to switch to cabozantinib. CT scans at this time were consistent with stable disease but AFP had increased from 7.4 to 11.9 ng/ml. Patient continued sorafenib 200 mg every other day until he started cabozantinib 40 mg by mouth (PO) QD in January 2020 with plans to titrate up to 60 mg as tolerated. The patient had diarrhea again despite use of anti-diarrheals as well as HFS despite use of urea-based creams. Cabozantinib was held in March 2020 with plans to restart at a lower dose of 20 mg every other day after resolution of diarrhea. Stool studies were performed to rule out infection and were negative. He was able to tolerate cabozantinib 20 mg PO every other day and was titrated back up to 20 mg QD, with minimal diarrhea and a good quality of life.

The patient received a liver transplant in April 2020, with explant showing residual disease in ablated lesions and confirming zero lymph node involvement (pN0). Cabozantinib was held prior to the transplant and continued to be held following transplant due to no evidence of disease. Patient is currently doing well with CT scans showing no evidence of new disease. We plan for close surveillance with clinic visits, labs, and CT scans every 3 months. He is currently 21 months out from transplant.

## Discussion

In the CELESTIAL study, 707 patients with HCC were randomly assigned to receive cabozantinib vs. placebo, and overall survival (OS), progression free survival (PFS), and objective response rate (ORR) were reported (16). These patients had previously received systemic treatment with sorafenib and had disease progression after at least one systemic treatment. Median OS was 10.2 months with cabozantinib vs. 8.0 months with placebo, median PFS was 5.2 months vs. 1.9 months, and ORR were 4% vs. <1% (16). There was, however, a 68% incidence of Grade 3 or 4 adverse events in the cabozantinib group vs. 36% in the placebo group (16). Both of our patients developed adverse effects, such as diarrhea and HFS at higher doses but tolerated cabozantinib at the lower 20 mg QD dose.

In our two patients, complications due to cabozantinib were not observed during the peri-transplant period. Symptoms of toxicities related to cabozantinib therapy were similar to toxicities observed in the CELESTIAL trial in patients with advanced HCC. Therefore, cabozantinib is a safe option after sorafenib failure or intolerance in patients with HCC awaiting liver transplant in whom bridge systemic therapy is considered.

We still have significant gaps in the literature regarding which patients receiving bridging therapy may have prolonged survival after transplant. For example, the first patient had early disease recurrence after transplant and died 11 months post-transplant despite continued systemic treatment after LT. Risk factors for recurrence include history of multifocal disease with multiple LRTs prior to transplant and significant residual disease on the explant. Currently, there is insufficient evidence to determine which patients with HCC would benefit from the use of cabozantinib or other agents (including immunotherapy) as a bridge to liver transplantation. However, as more data emerges in regard to the role of immunotherapy peri-transplant, immunotherapy and more novel agents, may be more effecting in downstaging HCC to increase transplant eligibility as well as controlling disease while patients await transplant. Therefore, future studies should prospectively investigate bridging therapy with newer agents in patients awaiting liver transplantation, so that we can optimize patient selection and increase eligibility for transplant.

## Data Availability Statement

The original contributions presented in the study are included in the article/supplementary material, further inquiries can be directed to the corresponding author/s.

## Author Contributions

All authors listed have made a substantial, direct, and intellectual contribution to the work and approved it for publication.

## Funding

This work was supported by the National Institutes of Health [Grant Number CA054174].

## Conflict of Interest

SA reports that she is on the speaker's bureau for Exelixis, Bristol-Meyers Squibb, and Bayer and advisory board for AstraZeneca and QED Therapeutics/Helsinn, and SA's institution has received research funding from Beigene, Faron, Halozyme, Ipsen, TerSera Therapeutics, Tvardi, and Caris Life Sciences. The remaining authors declare that the research was conducted in the absence of any commercial or financial relationships that could be construed as a potential conflict of interest.

## Publisher's Note

All claims expressed in this article are solely those of the authors and do not necessarily represent those of their affiliated organizations, or those of the publisher, the editors and the reviewers. Any product that may be evaluated in this article, or claim that may be made by its manufacturer, is not guaranteed or endorsed by the publisher.
